# Supplementary material for: Modeling glioblastoma heterogeneity as a dynamic network of cell states
Source: Mol Syst Biol. 2021 Sep 16;17(9):e10105. doi: 10.15252/msb.202010105 (PMC8444284; doi:10.15252/msb.202010105)
Supplement: Supplementary file 6 — Source Data for Figure 5 [file MSB-17-e10105-s004.zip › Figure5A_sourcedata/GSEA_3017/hallmarks_stateE.GseaPreranked.1621934677158/HALLMARK_SPERMATOGENESIS.html]

Details for gene set HALLMARK\_SPERMATOGENESIS[GSEA]

|  || Dataset | state63017 |
| Phenotype | NoPhenotypeAvailable |
| Upregulated in class | na\_neg |
| GeneSet | HALLMARK\_SPERMATOGENESIS |
| Enrichment Score (ES) | -0.61061156 |
| Normalized Enrichment Score (NES) | -2.1177237 |
| Nominal p-value | 0.0 |
| FDR q-value | 7.2673993E-4 |
| FWER p-Value | 0.005 |
Table: GSEA Results Summary

  

Fig 1: Enrichment plot: HALLMARK\_SPERMATOGENESIS      
 Profile of the Running ES Score & Positions of GeneSet Members on the Rank Ordered List

  

| PROBE | GENE SYMBOL | GENE\_TITLE | RANK IN GENE LIST | RANK METRIC SCORE | RUNNING ES | CORE ENRICHMENT || 1 | GSTM3 |  |  | 257 | 0.259 | -0.2506 | No |
| 2 | SLC12A2 |  |  | 389 | -0.279 | -0.3638 | No |
| 3 | PARP2 |  |  | 609 | -0.362 | -0.5634 | No |
| 4 | TOPBP1 |  |  | 614 | -0.364 | -0.5316 | No |
| 5 | NEK2 |  |  | 628 | -0.374 | -0.5085 | No |
| 6 | TTK |  |  | 724 | -0.454 | -0.5656 | Yes |
| 7 | GAD1 |  |  | 737 | -0.461 | -0.5328 | Yes |
| 8 | RFC4 |  |  | 777 | -0.527 | -0.5224 | Yes |
| 9 | AURKA |  |  | 824 | -0.623 | -0.5101 | Yes |
| 10 | CDKN3 |  |  | 836 | -0.653 | -0.4571 | Yes |
| 11 | BUB1 |  |  | 837 | -0.653 | -0.3923 | Yes |
| 12 | NCAPH |  |  | 845 | -0.685 | -0.3319 | Yes |
| 13 | KIF2C |  |  | 855 | -0.721 | -0.2701 | Yes |
| 14 | CCNB2 |  |  | 865 | -0.745 | -0.2059 | Yes |
| 15 | EZH2 |  |  | 886 | -0.810 | -0.1471 | Yes |
| 16 | DBF4 |  |  | 896 | -0.864 | -0.0711 | Yes |
| 17 | CDK1 |  |  | 938 | -1.248 | 0.0086 | Yes |
Table: GSEA details [plain text format]

  

Fig 2: HALLMARK\_SPERMATOGENESIS: Random ES distribution      
 Gene set null distribution of ES for **HALLMARK\_SPERMATOGENESIS**
